# An Unusual Iatrogenic Complication of Botulinum Toxin: Forearm Flexor Weakness Following a Nonstandard Intradermal Sensitivity Test

**DOI:** 10.1093/asjof/ojag132

**Published:** 2026-08-12

**Authors:** Ahmad Fayez Ahmad

## Abstract

Botulinum toxin (BoNT) type A is widely used in aesthetic and functional medicine and is generally considered safe when administered at appropriate doses and using proper techniques. However, rare and unusual complications may occur, even in patients with no previous neurological or allergic history. The author reports the case of a 24-year-old female who developed an unexpected neuromuscular complication characterized by progressive weakness of the forearm flexor muscles following a nonstandard intradermal sensitivity test of BoNT performed on the volar aspect of the forearm. The procedure was carried out by a nonspecialist practitioner. The patient subsequently developed reduced grip strength and functional limitation of hand movements within days, without systemic symptoms or clinical evidence of an allergic reaction. Clinical evaluation suggested a pharmacological neuromuscular effect rather than an immunologically mediated hypersensitivity reaction. No cutaneous signs of allergy, such as urticaria or angioedema, were observed. The temporal relationship and anatomical distribution of weakness were consistent with possible local toxin diffusion or unintended neuromuscular junction blockade. The patient was managed conservatively; a gradual improvement in muscle strength was observed over time. This case highlights a rare and atypical complication associated with the nonstandard use of BoNT and emphasizes the importance of adherence to evidence-based injection protocols performed by qualified specialists. It also raises concerns regarding the practice of intradermal “sensitivity testing,” which lacks scientific validation and may lead to unintended neuromuscular effects, particularly when performed by nonspecialists. Further studies are needed to clarify the safety implications of nonstandard applications and to distinguish true hypersensitivity reactions from pharmacologic diffusion-related effects.

Level of Evidence: 5 (Therapeutic)

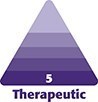

Botulinum toxin (BoNT), commonly known as Botox, is a neurotoxic protein produced by *Clostridium botulinum*, a gram-positive bacterium. The term “Botox” is derived from Botulinum TOXin and is commonly abbreviated as BoNT or BTX.

For therapeutic use, the toxin must be isolated, purified, and stabilized. BoNT comprises 7 distinct neurotoxin serotypes designated A, B, C (C1-C2), D, E, F, and G. These serotypes differ in serological and antigenic properties while sharing a similar structural framework. Serotypes A, B, and E are responsible for human foodborne botulism. Among them, Types A and B exhibit greater biological activity in living tissues, with effects lasting from several weeks to months, whereas Types C and D primarily affect animals.^1^

The use of BoNT may be associated with various complications, particularly when administered by inexperienced practitioners, when patients fail to adhere to postprocedural instructions, or when inaccurate dosages are used. Headache, including migraine, is the most commonly reported adverse effect, followed by local cutaneous reactions, such as ecchymosis or hematoma at the injection site, and neuromuscular symptoms.^2,3^

Other reported adverse effects include respiratory, ocular, cardiovascular, and gastrointestinal manifestations. Additionally, distant cutaneous reactions, facial asymmetry, and systemic symptoms, such as fatigue, have also been documented.^4^

Written informed consent for publication, including the use of clinical photographs, was obtained from the patient before submission. Institutional ethical exemption for this single case report was obtained in accordance with local institutional requirements. The study was conducted in compliance with the Declaration of Helsinki.

## CASE PRESENTATION

**Figure 1.**
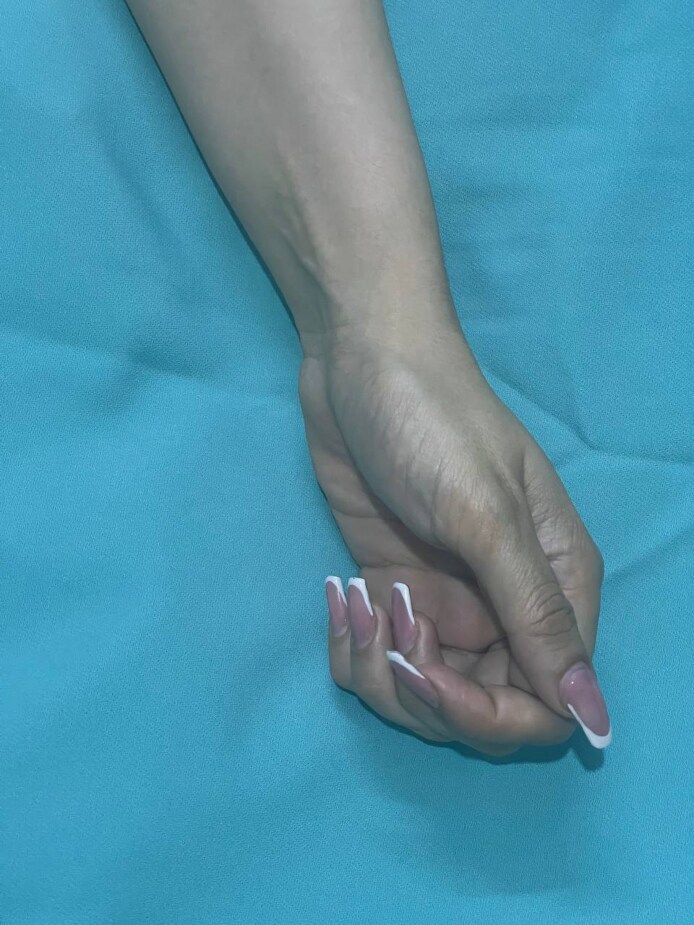
Clinical presentation of a 24-year-old female showing left-hand posture at initial assessment, demonstrating functional impairment with reduced grip and finger flexion (flexor digitorum superficialis, flexor digitorum profundus, flexor carpi radialis, and flexor carpi ulnaris).

This case was evaluated between August 2025 and March 2026. A 24-year-old female presented with discomfort and a subjective reduction in voluntary grip strength in the left hand Figure 1, which illustrates the clinical presentation, including hand posture and visible muscle involvement. Her medical history was unremarkable.

Three days before presentation, she had undergone a cosmetic facial BoNT injection performed by a nonspecialist practitioner outside our institution. Before the therapeutic injection, a nonstandard intradermal sensitivity test for BoNT was also performed on the volar aspect of the left forearm by the same practitioner—an approach that incorrectly applies allergy-testing paradigms used for other injectable agents to BoNT, for which no validated intradermal skin test exists. Complete procedural details were unavailable for independent verification; specifically, the commercial formulation, exact dose in units, dilution protocol, injection volume, needle gauge and length, precise intradermal depth, number of injection points, and distance from deeper muscular compartments could not be confirmed. The product reportedly used was Neuroxin; however, the batch number and storage conditions could not be verified. Although the injection was reported to the patient as intradermal, inadvertent deeper placement into subcutaneous or intramuscular tissue cannot be excluded.

Approximately 12 h after this intradermal exposure, the patient developed progressive weakness affecting grip strength and fine motor control of the fingers. Clinical examination by a neuromuscular specialist demonstrated weakness involving the forearm flexor muscles, particularly the flexor digitorum superficialis (FDS) and flexor digitorum profundus (FDP), as well as the wrist flexors—the flexor carpi radialis (FCR) and flexor carpi ulnaris (FCU). These muscles are essential for grip strength and finger flexion. Objective grip strength was measured using a hand dynamometer and was found to be markedly reduced compared with the contralateral side. No systemic or cutaneous allergic manifestations were observed.

The temporal relationship between exposure and symptom onset suggested possible localized diffusion of BoNT, resulting in unintended pharmacological neuromuscular junction blockade. Although the intended injection was intradermal, inadvertent deeper placement beyond the dermal layer represents a recognized technical risk, particularly among nonspecialist practitioners. Subcutaneous or intramuscular deposition of even small doses of BoNT in proximity to the flexor compartment muscles could plausibly account for the observed weakness through direct pharmacological effect at the neuromuscular junction. The anatomical proximity of the volar forearm flexor compartment to the skin surface—typically only several millimeters in depth—means that an inadvertently deep injection could reach muscular tissue. BoNT is known to diffuse within tissue in a dose- and volume-dependent manner, and even small intramuscular doses may produce clinically significant weakness in adjacent muscle groups.^1,2^

Electromyography (EMG) was performed 5 days after symptom onset. The muscles evaluated included the FDS, FDP, FCR, and FCU of the left forearm. Nerve conduction studies demonstrated normal sensory and motor conduction velocities with preserved compound muscle action potentials, arguing against a primary axonal or demyelinating neuropathy. No spontaneous denervation activity (fibrillation potentials or positive sharp waves) was identified. Motor unit action potential morphology was normal. The principal finding was reduced voluntary motor unit recruitment in the affected forearm flexor muscles without evidence of primary neurogenic or myopathic changes. Repetitive nerve stimulation was not formally performed. This pattern of reduced recruitment in the absence of denervation or neuropathic changes is consistent with presynaptic neuromuscular junction blockade as seen with BoNT. The absence of denervation potentials—which would be expected in axonal injury but not in pure neuromuscular junction (NMJ) blockade—further supports this interpretation.

### Differential Diagnosis

Although the temporal relationship between the intradermal injection and symptom onset is suggestive, causality cannot be established with certainty in a single case report without histological or pharmacological confirmation. The following alternative diagnoses were systematically considered.

Functional neurological disorder: The patient's symptoms were objective and reproducible on repeated clinical examination, with measurable grip strength deficit confirmed by dynamometry, making a purely functional etiology unlikely.

Compression neuropathy: Selective involvement of multiple forearm flexor muscles without sensory deficit and normal nerve conduction velocities argued against median or ulnar nerve compression neuropathy at the elbow or wrist.

Local traumatic injection injury: Traumatic injury from needle insertion alone could theoretically produce focal pain and limited weakness; however, it would not be expected to cause the diffuse pattern of forearm flexor involvement observed across multiple muscle groups.

Psychogenic weakness: The presence of consistent, reproducible, effort-independent weakness on examination and objective dynamometry measurement reduced the likelihood of psychogenic etiology, although it cannot be entirely excluded.

Early inflammatory neuropathy: Acute inflammatory conditions such as multifocal motor neuropathy or Parsonage–Turner syndrome were considered; however, the absence of pain typical of neuralgic amyotrophy, normal nerve conduction studies, and the absence of denervation potentials on EMG argued against these diagnoses.

Coincidental neuromuscular disorder: The abrupt onset immediately following the injection, with complete resolution within 45 days, makes a coincidental preexisting neuromuscular condition unlikely.

On balance, BoNT diffusion with inadvertent deeper placement was considered the most plausible explanation, although we acknowledge this remains a presumed etiology.

### Management and Outcome

Following discussion of management options, the patient was treated with oral pyridostigmine 30 mg every 8 h for 1 week, together with oral vitamin B complex supplementation once daily for 1 month. Structured physiotherapy and hand rehabilitation were also initiated. Pyridostigmine, an acetylcholinesterase inhibitor, was selected to augment acetylcholine availability at the neuromuscular junction with the aim of theoretically partially counteracting the presynaptic blockade induced by BoNT.^3^

It is acknowledged that spontaneous recovery would be expected over weeks to months as BoNT effect wanes following axonal sprouting and reinnervation. Therefore, the observed clinical improvement cannot be attributed solely to pyridostigmine, and its independent therapeutic efficacy in this context remains unproven. References supporting pyridostigmine use in NMJ blockade are limited; its use here was based on the theoretical rationale of enhancing cholinergic neurotransmission rather than proven clinical evidence in BoNT-related weakness.

Over 2 weeks, the patient showed marked clinical improvement, and complete resolution of symptoms was achieved within 45 days, with full restoration of hand function confirmed by dynamometry.

## DISCUSSION

This case describes a rare complication associated with BoNT type A (BoNT-A) in a patient with no previous history of allergic disease who initially presented for aesthetic treatment. To our knowledge, reports of focal forearm flexor weakness following intradermal BoNT sensitivity testing are extremely limited in the published literature.^1-4^

According to the US FDA prescribing information for onabotulinumtoxinA, pretreatment skin sensitivity testing is neither recommended nor required. Clinical use is based on appropriate patient selection, accurate dosing, and correct injection technique rather than allergy testing.^5^

Muner et al reported a case of a 50-year-old female who developed a localized inflammatory reaction characterized by erythema, edema, vesicle formation, burning sensation, and pruritus following upper facial injections. A provocation test using a different formulation injected into subcutaneous tissue showed a stronger response, suggesting higher immunological reactivity in subcutaneous tissue compared with muscle. Standard corticosteroid and antibiotic therapy was ineffective, whereas ozone therapy resulted in marked improvement.^6^

Buchholz et al presented 3 cases suggestive of hypersensitivity reactions to BoNT-A. In the first case, a 44-year-old woman developed severe angioedema after resuming abobotulinumtoxinA; intradermal testing demonstrated localized reactions, suggesting a shared antigenic component across formulations. In the second case, a 38-year-old woman developed delayed erythema and swelling after letibotulinumtoxinA but tolerated incobotulinumtoxinA without reaction, suggesting a role of accessory proteins or excipients. In the third case, a 50-year-old woman developed systemic symptoms after prabotulinumtoxinA, consistent with delayed-type hypersensitivity.^7^

Glass et al described a female patient treated with intrapalmar injection of BoNT-A for hyperhidrosis who subsequently developed atrophy and functional weakness of the intrinsic hand muscles after repeated treatment sessions. Clinical assessment and nerve conduction studies demonstrated bilateral muscle weakness with functional changes in the thenar and interosseous muscles, without any other clear neurological or systemic causes. Some parameters improved after discontinuation of treatment and rehabilitation, although partial weakness persisted, raising the possibility of an adverse effect associated with repeated intrapalmar use of BoNT-A.^8^

### Limitations

The principal limitation of this report is the absence of complete procedural details, because the injection was performed by a third-party nonspecialist practitioner outside our institution. Information regarding the exact dose, dilution, injection volume, needle specifications, depth of injection, number of injection points, and distance from deep muscular compartments was unavailable. Without these data, it is not possible to fully evaluate the plausibility of sufficient toxin diffusion to affect the forearm flexors. Additionally, this is a single case report and therefore cannot establish causality; histological or pharmacological confirmation of BoNT activity in the affected muscles was not obtained. Repetitive nerve stimulation was not performed, which could have provided additional electrophysiological support for presynaptic NMJ blockade. The independent contribution of pyridostigmine to recovery cannot be determined given the expected natural history of BoNT-related weakness. Finally, this report is limited to a single institution and a single patient, restricting generalizability.

Despite these limitations, this case contributes to the published literature by documenting a rare and clinically important complication of BoNT use outside the setting of a specialist practice. It reinforces existing regulatory guidance against nonstandard sensitivity testing and adds to the body of evidence regarding the risks associated with inadvertent deep injection by nonspecialist practitioners. The systematic differential diagnosis, objective grip strength documentation, and detailed EMG findings enhance the scientific rigor of this report relative to previous case descriptions.

## CONCLUSIONS

This case highlights a rare complication associated with BoNT-A that, to our knowledge, has been extremely rarely described in the published literature. It occurred in a patient without previous allergic history and was successfully managed conservatively without surgical intervention. The complication is likely related to the increasing use of aesthetic procedures performed by nonspecialist practitioners, and the exact mechanism—whether because of inadvertent deep injection or pharmacological diffusion—could not be definitively established from the available information.

This case underscores the importance of patient education and reinforces that all medical and aesthetic procedures involving BoNT should be performed exclusively by qualified and experienced specialists. Furthermore, intradermal sensitivity testing for BoNT is not endorsed by regulatory guidelines and should be actively discouraged.
